# Bridging the marrow: a co-culture-platform of leukemia cells and MS5-derived stromal cells or adipocytes

**DOI:** 10.1038/s41420-025-02631-5

**Published:** 2025-08-05

**Authors:** Julia Zinngrebe, Elena Dorothea Brenner, Ferdinand Schlichtig, Ulrich Stifel, Daniel Tews, Jana Falk, Dominik Schlotter, Rahel Fitzel, Lüder-Hinrich Meyer, Klaus-Michael Debatin, Pamela Fischer-Posovszky

**Affiliations:** 1 Department of Pediatrics and Adolescent Medicine, Ulm University Medical Center, Ulm, Germany; 2 Division of Pediatric Endocrinology and Diabetes, Department of Pediatrics and Adolescent Medicine, Ulm University Medical Center, Ulm, Germany; 3 German Center for Child and Adolescent Health (DZKJ), partner site Ulm, Ulm, Germany

**Keywords:** Paediatric cancer, Cancer microenvironment, Leukaemia, Experimental models of disease

## Abstract

In the context of acute lymphoblastic leukemia (ALL), the bone marrow microenvironment (BMM) plays a crucial role in providing pro-survival signals, as evident from the rapid spontaneous cell death observed in ex-vivo cultures of primary ALL cells. Among the diverse cell types within the BMM, bone marrow adipocytes (BMAd) exhibit significant plasticity and can constitute a substantial part of the BMM, especially during ALL therapy. However, conflicting data on the interaction between ALL cells and adipocytes have been reported, potentially arising from variations in culture systems and conditions. Our study aimed to establish a chemically defined co-culture system of leukemia cells combined with either bone marrow (BM)-derived stromal cells or adipocytes. Established B-precursor ALL cell lines, patient-derived ALL xenografts (PDX), and murine BM-derived MS5 stromal cells and adipocytes were used as model systems. Fetal calf serum and factors included in the adipogenic media significantly impacted leukemia cell viability and proliferation. Thus, we implemented a washing procedure and suitable, chemically defined media conditions into our co-culture platform. In general, ALL cell lines survived and proliferated on both stromal cells and adipocytes, whereas PDX cells exhibited a pronounced survival advantage on stromal cells compared to adipocytes. Intriguingly, the presence of adipocytes sensitized cell lines and PDX cells to chemotherapy with anthracyclines or dexamethasone when compared to co-cultures with stromal cells. Thus, utilizing the well-established MS5 cell line together with the optimized culture conditions in our co-culture system provides a reliable, open-access platform for investigating intricate interactions between bone marrow stromal cells, adipocytes, and leukemia cells.

## Introduction

Acute lymphoblastic leukemia (ALL) is the most prevalent cancer in childhood [1] and originates in the bone marrow (BM). Cure rates reach up to 90% but prognosis worsens drastically in case of relapse, particularly in those patients with BM infestation [2]. The bone marrow microenvironment (BMM) is formed by a variety of different cell types, amongst them cells of the hematopoietic system and mesenchymal stem cells (MSCs), which can further differentiate into other cell types, e.g., osteoblasts, chondrocytes, and adipocytes. The BMM influences the survival and proliferation of leukemic blasts. This is highlighted by the fact that primary B-precursor ALL cells rapidly undergo spontaneous cell death when cultured outside their familiar surroundings in the BMM ex vivo [3]. Thus, interference with the BMM’s pro-survival signals is a promising strategy in the treatment of ALL.

The role of adipocytes in ALL is complex. BM-derived adipocytes (BMAd) show an enormous plasticity and can form a major part of the BMM under certain conditions, for example, in BM of patients diagnosed with B-precursor ALL at the end of induction chemotherapy [4–6]. Noteworthy, fasting, a condition known to result in increased BMAd in mice [7], inhibited leukemia development and progression in mouse models of B-ALL and T-ALL, respectively [8]. In line with this, the presence of adipocytes in the murine BM interferes with engraftment of human T-ALL [9] and B-precursor ALL [6] to these BM sites in vivo, suggesting that BMAd plays a tumor-suppressive role in leukemogenesis. On the contrary, several studies by Mittelman and colleagues show that adipocytes can protect ALL cells from chemotherapy-induced cytotoxicity in vivo and in vitro [5, 10–15].

A variety of different culture conditions and different cell strains with adipogenic differentiation capacity have been used in the relevant literature [5, 6, 9, 11–15], complicating data comparison. This led us to establish a leukemia cell-adipocyte co-culture system to assess the influence of BMAd on survival, proliferation, and response to chemotherapy of B-precursor ALL cell lines in vitro and patient-derived B-precursor ALL xenografts ex vivo. Here, we provide data on the successful differentiation of BM-derived MS5 stromal cells into adipocytes and the impact of adipogenic factors and FCS on leukemia cell viability. By using our co-culture platform, we compared how stromal cells versus adipocytes impacted leukemia cell proliferation, survival, and sensitivity to chemotherapy.

## Results

### The bone marrow-derived MS5 stromal cell line possesses robust adipogenic differentiation capacity

**Fig. 1:**
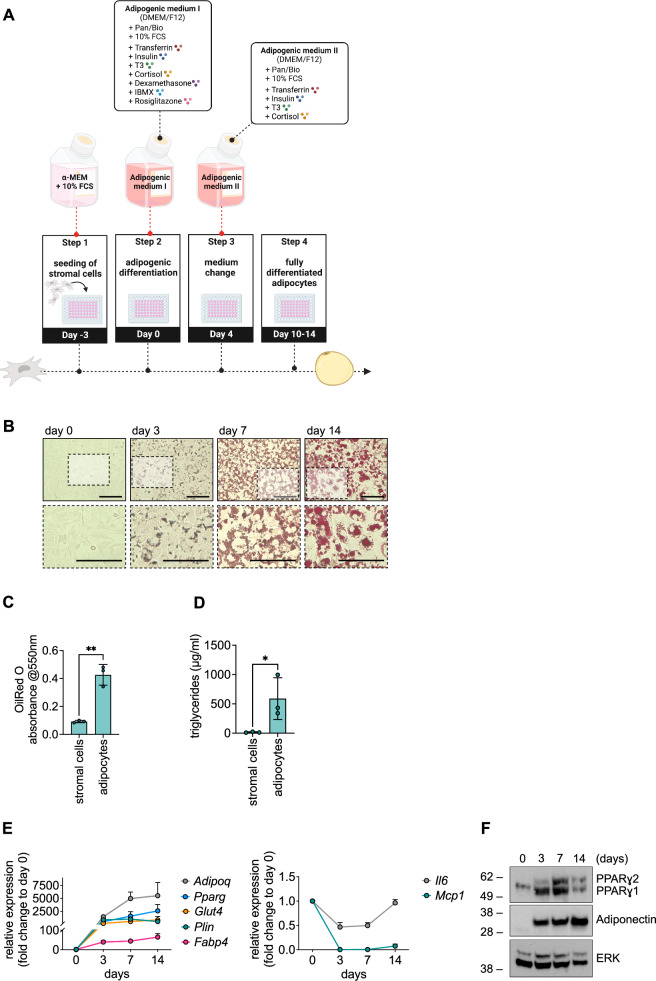
The bone marrow-derived MS5 stromal cell line possesses robust adipogenic differentiation capacity. **A** Protocol of adipogenic differentiation is depicted. **B** MS5 cells were stained with Oil Red O on different days of adipogenic differentiation and representative microphotographs are shown. Scale bars equate to 100 µm. **C** and **D** Oil Red O absorbance (**C**) and triglycerides (**D**) were determined in MS5 stromal cells (day 0 of adipogenic differentiation) and adipocytes (day 9 of adipogenic differentiation). Data are presented as mean $\pm \,$ SD of three independent experiments performed in triplicates. Unpaired *t*-test, *P < 0.05; **P < 0.01. **E** and **F** Expression of adipogenic markers on mRNA (**E**) and protein (**F**) levels during the course of adipogenic differentiation is shown. Data are presented as mean ± SEM of at least three independent experiments performed in duplicates (**E**). Images are representative of three independent experiments (**F**).

Isolation of primary human or murine BM-derived MSCs is time-consuming, and material is limited. A human BM-derived cell line with adipogenic differentiation capacity does not exist [16]. To establish a ready-to-use adipocyte–leukemia cell co-culture system, we assessed the adipogenic differentiation capacity of the murine BM-derived stromal cell line MS5 [9, 17, 18]. Using a known adipogenic differentiation protocol [19, 20] (Fig. 1A), MS5 cells rapidly incorporated droplets filled with lipids (Fig. 1B–D). These droplets covered about 40% of the cellular surface from day 5 of adipogenic differentiation onwards (Supplementary Fig. S1A). The change in morphology was accompanied by an upregulation of adipocyte-specific markers on mRNA (Fig. 1E) and protein level (Fig. 1F). Of note, whilst adipogenic marker genes increased during adipogenic differentiation in MS5 cells (Fig. 1E, left panel), inflammatory genes such as *Il6* and *Mcp1* decreased (Fig. 1E, right panel).

The murine 3T3-L1 cell line, derived from subcutaneous white adipose tissue (scWAT), is known for its robust adipogenic differentiation capacity (Supplementary Fig. S1B–E) and has been widely used in the leukemia field [5, 6, 11–15]. Comparative analysis revealed slight differences in the expression levels of typical adipogenic markers on the mRNA and protein levels between scWAT-derived 3T3-L1 cells and BM-derived MS5 cells (Supplementary Fig. S1F and G), in line with previous reports on differential gene expression in adipocytes derived from bone marrow and WAT depots [21–24].

We conclude that BM-derived MS5 cells possess adipogenic differentiation capacity and can be effectively transformed into adipocytes using an established protocol of adipogenic differentiation. To study the interaction of leukemia cells with BM-derived stromal cells and adipocytes, MS5 cells may therefore represent a suitable model system.

### Adipogenic factors influence viability of B-precursor ALL cells

**Fig. 2:**
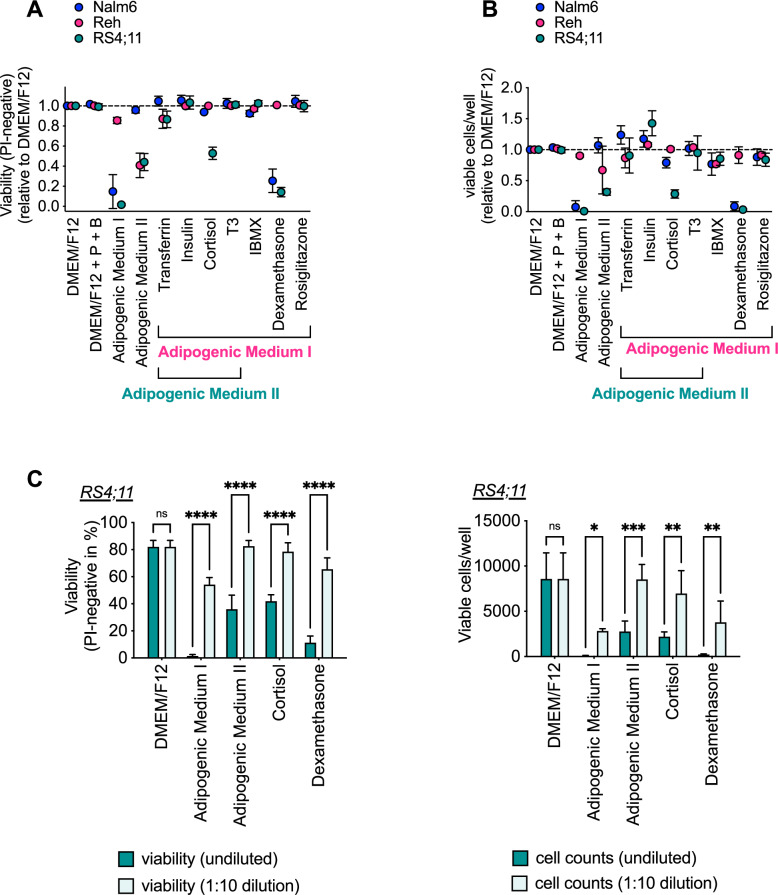
Adipogenic factors influence the viability of B-precursor ALL cells. **A** and **B** B-precursor ALL cell lines Nalm6, Reh, and RS4;11 were incubated with adipogenic differentiation factors alone or in combination. After 96 h, viability (**A**) and the number of viable cells per well (**B**) were determined by FACS. P = pantothenate, B = biotin. **C** The B-precusor ALL cell line RS4;11 was incubated with adipogenic differentiation factors, undiluted or diluted 1:10. Viability and the number of viable cells per well were determined after 96 h by FACS. Data is presented as mean ± SD of at least three independent experiments performed in triplicates. *P < 0.05; **P < 0.01; ***P < 0.001; ****P < 0.0001; two-way ANOVA with Bonferroni’s multiple comparisons test.

Co-culture of different cell types is hampered by different culturing requirements. Adipogenic factors are indispensable for the induction of adipogenic differentiation. These factors include glucocorticoids such as cortisol or dexamethasone, which, due to their cytotoxicity on lymphoid cells, have been an essential component of therapeutic regimens for B-precursor ALL for years [25, 26]. Thus, we determined how the different adipogenic factors alone or in combination affect viability (Fig. 2A) and proliferation (Fig. 2B) of different B-precursor ALL cell lines. Adipogenic Medium I and II affected the viability (Fig. 2A) of the three cell lines Nalm6, Reh or RS4;11 to different extents. This reduction in viability was accompanied by a reduction in the total number of viable cells per well (Fig. 2B). Reduction of the adipogenic factors’ concentration in the medium by one tenth already resulted in significantly increased viability of RS4;11 cells (Fig. 2C). Thus, adipogenic factors represent a potential confounder in a co-culture system of leukemic cells and adipocytes that should be controlled for.

### Establishment of a serum-free and chemically defined leukemia cell-adipocyte co-culture system

**Fig. 3:**
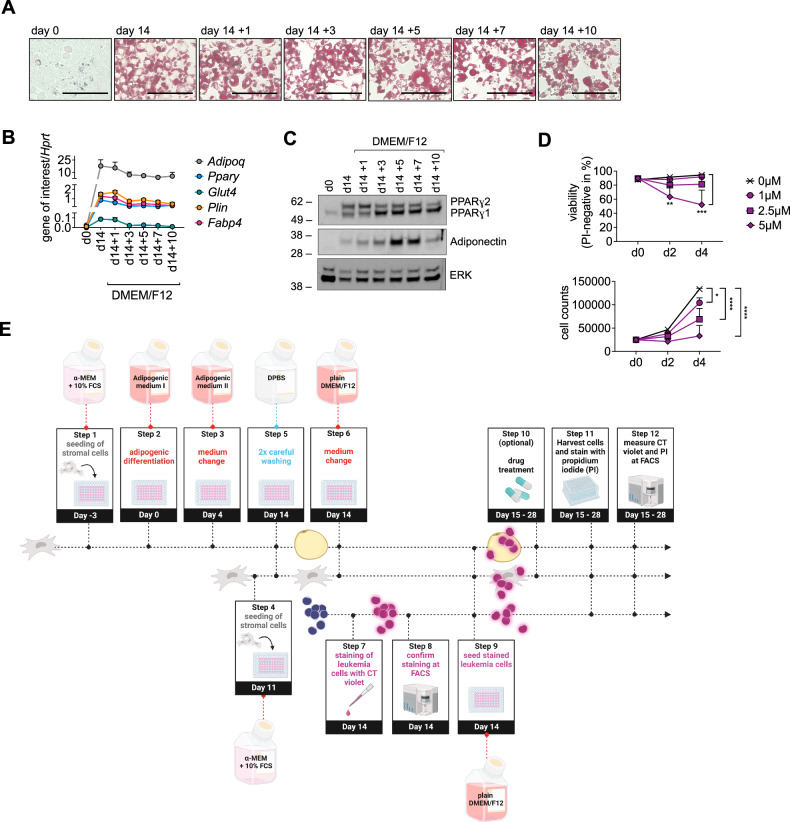
Establishment of a serum-free and chemically defined leukemia cell–adipocyte co-culture system. **A**–**C** MS5 cells were stained with Oil Red O (**A**) and assessed for expression of adipogenic markers on mRNA (**B**) and protein (**C**) level on days 0 and 14 of adipogenic differentiation and on following days after medium change to DMEM/F12 on day 14 (d14 + 1 to +10). Scale bars equate to 100 µm (**A**). Data are presented as mean ± SEM of at least three independent experiments performed in triplicates (**B**). Images are representative of three independent experiments (**C**). **D** The leukemia cell line RS4;11 was labeled with increasing concentrations of CellTrace Violet (CT) as indicated. Viability and the number of viable cells per well were assessed after 2 and 4 days by propidium iodide (PI) staining at the FACS. Data are presented as mean ± SEM of three independent experiments performed in triplicates. *P < 0.05; **P < 0.01; ***P < 0.001; ****P < 0.0001; two-way ANOVA with Tukey’s multiple comparisons test. **E** Schematic illustration of the serum-free and chemically defined leukemia cell–adipocyte co-culture system.

Adipogenic factors are crucial for adipogenic differentiation. However, once fully differentiated, adipocytes might be able to survive without them. To remove residual adipogenic factors from the culture medium, we performed two washing steps of fully differentiated adipocytes on day 14 of adipogenic differentiation. Fully differentiated adipocytes are very fragile. Therefore, washing of adipocytes should be performed carefully. To control for possible cytotoxic effects of the washing procedure and the removal of adipogenic factors, we evaluated the morphology of the cells by Oil Red O staining on different days following medium change (Fig. 3A). Of note, the cells retained their typical adipocyte appearance containing lipid droplets up to day 10 after medium change. In the next step, we assessed how the removal of adipogenic factors influenced the expression of adipogenic markers at the mRNA and protein levels. After washing and medium change, adipocytes still expressed adipocyte-specific marker genes (Fig. 3B) and proteins (Fig. 3C). To discriminate leukemia cells from adipocytes and stromal cells during co-culture, we labeled the leukemic cells with CellTrace Violet (CT). Whereas higher concentrations of CT were toxic to leukemia cells, lower concentrations of CT did not affect viability and only slightly influenced proliferation of the B-precursor ALL cell line RS4;11 (Fig. 3D). Using CT at a concentration of 1 µM allowed discrimation of ALL cells from both, stromal cells and adipocytes for up to 14 days in co-culture (Supplementary Fig. S2). We integrated our findings into a step-by-step protocol describing the establishment of a serum-free and chemically defined leukemia cell–adipocyte co-culture system (Fig. 3E).

### BM-derived stromal cells and adipocytes maintain survival and proliferation of B-precursor ALL cell lines

**Fig. 4:**
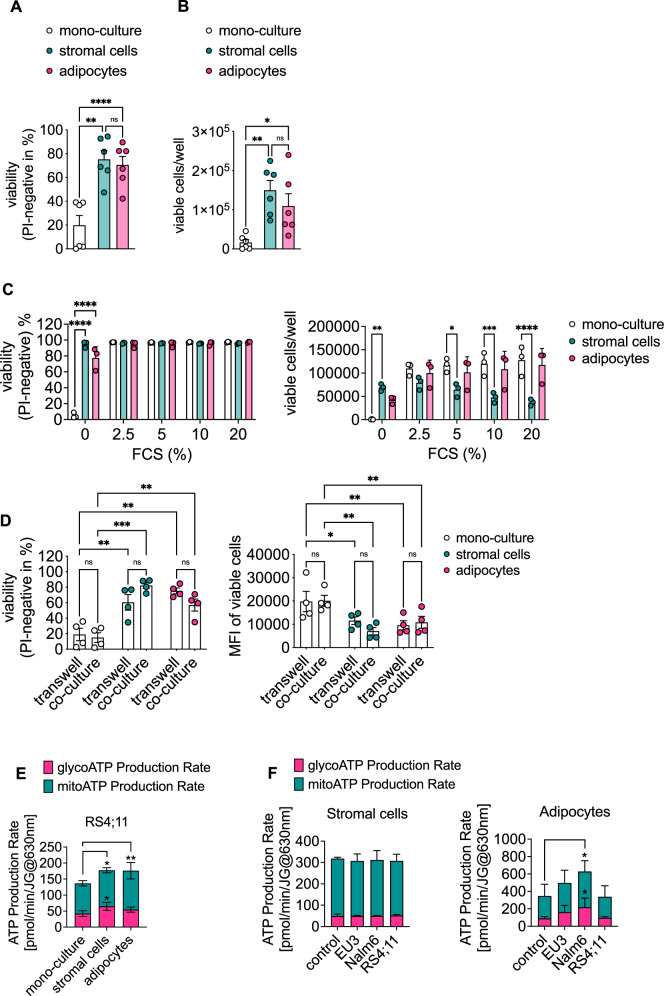
MS5-derived stromal cells and adipocytes maintain the survival and proliferation of B-precursor ALL cell lines. **A** and **B** B-precursor ALL cell lines EU3, KOPN-8, RCH-ACV, Nalm6, Reh, and RS4;11 (each dot represents one cell line) were cultured alone or together with stromal cells or adipocytes derived from MS5 cells. Cell viability (**A**) and the number of viable cells per well (**B**) were determined after 7 days by FACS. **C** EU3 mono-culture was compared to co-culture with MS5-derived stromal cells or adipocytes cultured in DMEM/F12 medium without FCS, compared to medium with increasing concentrations of FCS as indicated. Viability and the number of viable cells per well were determined by FACS analysis after 7 days. **D** The B-precursor ALL cell line RS4;11 was labeled with CellTrace Violet (CT) and cultured alone or together with MS5-derived stromal cells or adipocytes, either directly or in transwell inserts. Viability and mean fluorescence intensity (MFI) of CellTrace Violet were determined on day 7 after seeding. **E** RS4;11 cells were co-cultured in transwell inserts over medium (mono-culture), MS5-derived stromal cells, or adipocytes for 72 h. Subsequently, ATP production was analyzed by the Seahorse Extracellular Flux Analyzer. **F** MS5-derived stromal cells (left panel) and adipocytes (right panel) were incubated with leukemia-conditioned media for 24 h and subsequently analyzed by the Seahorse Extracellular Flux Analyzer. ATP production rates are depicted. Data are presented as mean ± SEM (**A**, **B**, **D**) or as mean ± SD (**C**, **E**, **F**) of at least three independent experiments performed in triplicate (**A**–**C**) or duplicates (**D**) or in three to five technical replicates (**E**, **F**). RM one-way ANOVA with Tukey’s multiple comparisons test (**A**, **B**), two-way ANOVA with Dunnett’s (**C**, **E**, **F**) or Tukey’s (**D**) multiple comparisons test, *P < 0.05; **P < 0.01; ***P < 0.001; ****P < 0.0001.

To investigate the extent to which adipocytes support survival or proliferation of B-precursor ALL cell lines under the established chemically defined culture conditions, we cultured six different B-precursor ALL cell lines, i.e. RS4;11, Nalm6, Reh, RCH-ACV, EU3, and KOPN-8, either alone or together with MS5-derived stromal cells or adipocytes. We analyzed cell viability (Fig. 4A) and cell proliferation as determined by the number of viable cells per well (Fig. 4B) after 7 days in culture. Of note, co-culture with BM-derived stromal cells and adipocytes significantly maintained survival (Fig. 4A) or stimulated proliferation of B-precursor ALL cell lines (Fig. 4B) as compared to mono-culture for 7 days. Interestingly, BM-derived stromal cells and adipocytes were equally efficient in doing so (Fig. 4A and B). Data of individual cell lines can be found in Supplementary Fig. S3A and B.

Of note, MS5 stromal cells and adipocytes were superior to 3T3-L1 stromal cells and adipocytes in maintaining the survival of B-precursor ALL cell lines EU3, Nalm6, and RS4;11 (Supplementary Fig. S5A and B). Furthermore, survival of B-precursor ALL cell lines was largely independent of the culture medium (Supplementary Fig. S6A and B) and the ratio of leukemia cell to stromal cell or adipocyte in the assay (Supplementary Fig. S7A and B).

A lot of published data were generated in co-culture experiments performed under serum-containing conditions [6, 9, 12, 14]. FCS, however, is a known disturbance variable and impacts data reproducibility [27]. The addition of even low concentrations of FCS to the culture resulted in leukemia cells surviving independently of feeder cell support since mono-cultures and co-cultures survived to similar extents in the presence of FCS (Fig. 4C).

This data illustrates how important well-controlled culture conditions are for the conduct of co-culture experiments.

Next, we determined whether the survival and proliferation of leukemia cells in co-culture with BM-derived stromal cells or adipocytes is maintained by direct cell–cell contact or via secreted factors. To do so, we performed an indirect co-culture of the leukemia cell line RS4;11 in transwell inserts preventing direct cell–cell-interaction but allowing secreted factors to pass via the membrane pores and compared it to direct co-culture (Fig. 4D). After 7 days in culture and as expected, viability of RS4;11 cells was significantly higher in both, direct and indirect co-cultures with BM-derived stromal cells or adipocytes as compared to mono-culture (Fig. 4D). Importantly, viability of leukemia cells cultured in direct versus indirect co-culture did not significantly differ. This indicates that the viability of leukemia cell lines is maintained by soluble factors, and not by direct cell–cell contact with stromal cells or adipocytes. In addition, proliferation of RS4;11 cells, as assessed by mean fluorescence intensity of CT, was supported by the presence of both stromal cells and adipocytes (Fig. 4D).

Next, we investigated whether the metabolism of B-precursor ALL cells is influenced by the presence of BM-derived stromal cells and/or adipocytes. Thus, RS4;11 cells were cultured in transwell inserts over medium, stromal cells or adipocytes, and subsequently analyzed after 96 h using the Seahorse Extracellular Flux Analyzer (Fig. 4E). Under the conditions chosen, the overall ATP production significantly increased in RS4;11 cells upon indirect co-culture with either stromal cells or adipocytes (Fig. 4E). This suggests that secreted factors or metabolites of stromal cells and adipocytes lead to increased energy production in leukemia cells needed for their survival and proliferation. Vice versa, we assessed whether leukemia cells affected the metabolism of stromal cells or adipocytes. To address this question further, we generated conditioned media of the B-precursor ALL cell lines Nalm6, EU3 and RS4;11, and incubated either stromal cells or adipocytes therein for 24 h (Fig. 4F). Overall, both cell types remained largely unaffected by the leukemia-conditioned media in terms of glycolytic and mitochondrial metabolism with one exception, i.e. adipocytes significantly increased their ATP production when stimulated with conditioned medium of Nalm6 cells (Fig. 4F). This increase in ATP levels in adipocytes could hint to a metabolic reprogramming of the adipocytes by leukemia-secreted factors. The data further suggests that a re-programming of BM adipocyte metabolism might occur depending on the biology of the particular leukemia. Therefore, some leukemia subtypes might be unaffected while others might benefit from the presence of BM adipocytes. To study this further, we analyzed the capacity of 24 individual, patient-derived B-precursor ALL samples to survive and proliferate in the presence of MS5-derived stromal cells or adipocytes.

### MS5-derived stromal cells are superior to adipocytes in maintaining survival and proliferation of PDX B-precursor ALL cells

**Fig. 5:**
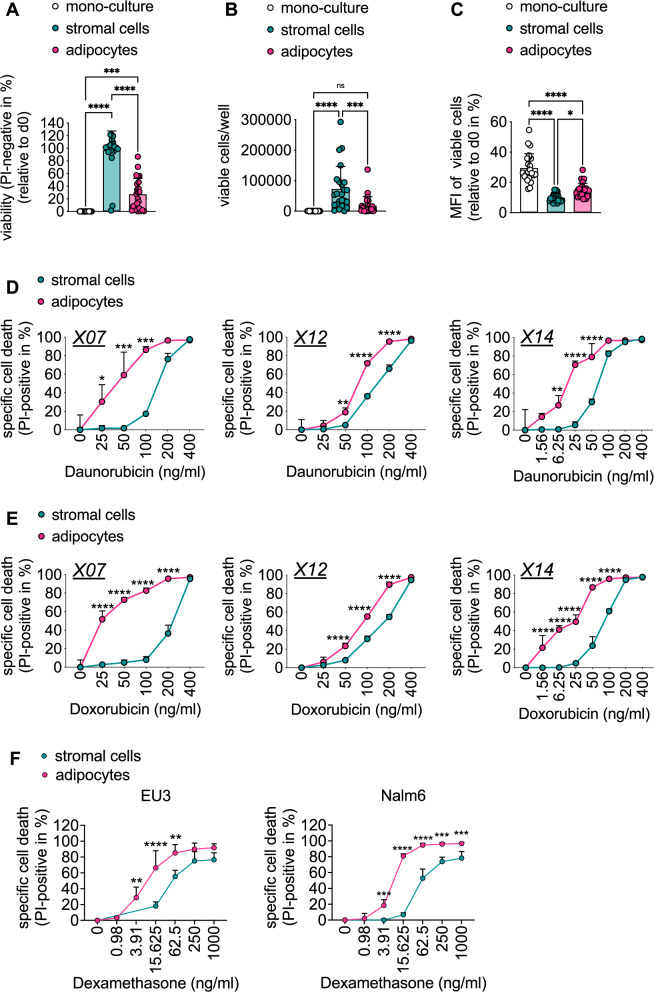
MS5-derived stromal cells are superior to adipocytes in maintaining survival and proliferation of PDX B-precursor ALL samples ex vivo, and in preventing PDX cells from chemotherapy-induced cytotoxicity. **A**–**C** PDX B-precursor ALL samples (*n* = 24) were cultured alone or together with MS5-derived stromal cells or adipocytes. Viability (**A**), the number of viable cells per well (**B**), and mean fluorescence intensity (MFI) of CellTrace relative to day 0 (**C**) were determined after 7 days. **D** and **E** Three different human PDX samples (X07, X12, X14) were stimulated with the indicated concentrations of daunorubicin (**D**) or doxorubicin (**E**), and PI positivity was measured after 7 days. **F** B-precursor ALL cell lines EU3 and Nalm6 were stimulated with increasing concentrations of dexamethasone as indicated in co-culture with either MS5-derived stromal cells or adipocytes. Cell death was assessed by PI positivity at the FACS after 96 h. Data are presented as mean ± SD of individual PDX B-precursor ALL samples represented by single dots measured in triplicate (**A**–**E**) or of three independent experiments performed in triplicates (**F**). *P < 0.05; **P < 0.01; ***P < 0.001; ****P < 0.001; ordinary one-way ANOVA with Tukey’s multiple comparisons test (**A**–**C**), two-way ANOVA with Šídák’s multiple comparisons test (**D**–**F**).

Primary B-precursor ALL cells do not grow without niche support and rapidly undergo spontaneous cell death ex vivo [3]. We therefore next assessed the extent to which MS5-derived stromal cells or adipocytes were able to support the survival and proliferation of different PDX B-precursor ALL samples ex vivo. Of note, under the conditions chosen herein, almost all PDX samples, apart from two, survived for a period of 7 days to almost 100% on MS5-derived stromal cells, whereas none of the PDX samples survived in mono-culture (Fig. 5A; see Supplementary Fig. S4A and B for viability on day 0 and survival of individual PDX samples over time). Interestingly, the survival of the PDX samples on adipocytes was heterogeneous and significantly lower as compared to co-culture on MS5-derived stromal cells (Fig. 5A). The number of viable cells per well paralleled with the viability data (Fig. 5B). To assess whether PDX B-precursor ALL cells also proliferated on the respective cell types, we determined the mean fluorescence intensity (MFI) of leukemia cells labeled with CT and found the dye to be significantly more reduced in cells derived from co-culture samples as compared to mono-culture (Fig. 5C). This is evidence of a washout of the dye indicative of proliferation. To correct for the variation of the MFI on day 0 in between samples (Fig. S4C), we show the MFI relative to day 0 for each sample (Fig. 5C). Importantly, the MFI remained significantly higher in co-cultures on adipocytes as compared to co-cultures on stromal cells, indicating that PDX cells proliferated less in the presence of adipocytes as compared to stromal cells (Fig. 5C).

This data suggests that the presence of adipocytes has a negative influence on the survival and proliferation of leukemia cells as compared to stromal cells, and that PDX cells show a strong heterogeneity with respect to their survival capacity on adipocytes.

In additional experiments, we compared the capacity of MS5-derived stromal cells or adipocytes with 3T3-L1-derived stromal cells or adipocytes in supporting the survival of PDX B-precursor ALL cells (Supplementary Fig. S5C and D). Here, we identified MS5 cells to be superior to 3T3-L1 cells in promoting the survival of PDX B-precursor ALL cells ex vivo (Supplementary Fig. S5C and D). Furthermore, PDX B-precursor ALL cells survived on MS5-derived stromal cells or adipocytes independently of the culture medium (Supplementary Fig. S6C and D) and independently of the ratio of leukemia cell to stromal cell or adipocyte (Supplementary Fig. S7C and D).

Adipocytes were shown to protect leukemia cells from the cytotoxic effects of chemotherapy [5, 12–15]. Moreover, adipocytes were identified to be able to metabolize the anthracycline daunorubicin [13]. Thus, to assess the drug response of leukemia cells in our serum- and adipogenic factor-free leukemia cell–adipocyte co-culture system, we next performed treatment with daunorubicin of three different leukemia cell lines in co-culture with either MS5-derived stromal cells or adipocytes (Fig. S8A, see Fig. S8C for viability of untreated leukemia cells). We were surprised to observe that the different leukemia cell lines remained equally sensitive to treatment with daunorubicin (as observed for the leukemia cell line Nalm6) or were even more sensitive to daunorubicin in the presence of adipocytes (as observed for EU3 and Reh cells) (Fig. S8A). To further validate this finding, we next treated PDX cells in co-culture with MS5-derived stromal cells or adipocytes with daunorubicin (Fig. 5D). Interestingly, the sensitization to daunorubicin-induced cell death in co-culture with adipocytes as compared to stromal cells was even more pronounced in PDX samples (Fig. 5D; see Fig. S8E for viability of untreated leukemia cells). The very same effect we observed in PDX samples treated with doxorubicin (Fig. 5E). Important controls showed viability of stromal cells or adipocytes was unaffected by daunorubicin (Fig. S8B) and doxorubicin (Fig. S8D) in the concentration range used in the experiments with leukemia cells. Therefore, the presence of adipocytes appears to elicit a sensitizing effect on anthracycline therapy in B-precursor ALL cell lines and PDX samples.

These data contradict published data and once again highlight the importance of using a well-controlled model system for thorough characterization of the role of adipocytes in ALL. Due to their cytotoxic potential, glucocorticoids such as dexamethasone have been an integral part of ALL therapy for decades. To assess whether the presence of adipocytes sensitized leukemia cells to anthracycline therapy only, we tested the effect of dexamethasone on the leukemia cell lines EU3 and Nalm6 cultured in presence of either MS5-derived stromal cells or adipocytes (Fig. 5F). Both, Nalm6 and EU3 cells were more sensitive to dexamethasone-induced cytotoxicity in the presence of adipocytes as compared to stromal cells (Fig. 5F; for toxicity of dexamethasone on stromal cells and adipocytes see Fig. S8F, for viability of untreated leukemia cells see Fig. S8G). This suggests that an increase in chemosensitivity of leukemia cells in the presence of adipocytes, as compared to stromal cells, is a more general phenomenon and not restricted to anthracyclines.

## Discussion

Our goal was to develop a co-culture system of leukemia cells and adipocytes derived from BM and their respective precursor cells, which meets the requirements of all cell types involved, especially allowing the examination of primary leukemia samples. Moreover, we aimed to establish a model system that is easily accessible to as many scientists as possible to ensure comparability of data from different laboratories. The use of primary human MSCs—apart from their limited availability—is associated with high variability [28]. Therefore, we resorted to the use of cell lines with adipogenic differentiation capacity. One additional important aspect was to establish media conditions with as few confounding variables as possible. Many protocols of adipogenic differentiation include steroids, whose cytoreductive potential for leukemia cells is all too well known and has been used in ALL therapy for decades [25]. Our data show that the steroid concentrations used during adipogenic differentiation are cytotoxic to ALL cell lines. Thus, adipogenic factors in general, but glucocorticoids in particular, should be avoided during the performance of co-culture experiments with adipocytes and ALL cells. In-vitro co-culture models of leukemia cells and adipocytes described in the literature often lack the information as to whether essential washing steps were performed before co-culture. In fact, we were only able to retrieve this information from the “Methods” section of one publication [6]. It seems essential that this information is provided to the reader to allow a classification and evaluation of the published results. Especially as we show that FCS is an additional potential confounding factor in the co-cultivation of stromal cells or adipocytes with ALL cells that should not be underestimated. In addition to the known influence of FCS on data reproducibility [27], may its use in this particular case mask effects mediated by the stromal cells or adipocytes and, for example, lead to a putatively lower proliferation rate of leukemia cell lines on stromal cells compared to mono-culture or co-culture on adipocytes (Fig. 4C).

Whether BM-derived stromal cells and adipocytes differentially regulate the survival and proliferation of ALL cells is still not entirely understood. Moreover, data on primary ALL cells in co-culture experiments with adipocytes ex vivo are scarce. For primary T-ALL samples, their survival was shown to be worse on adipocytes than on the associated MSCs [9]. Thus, another aim of our work and the establishment of this co-culture system was to allow direct comparability of BM-derived stromal cells and adipocytes to assess their potentially differential influence on survival and proliferation of leukemia cell lines in vitro and PDX cells ex vivo. Both BM-derived stromal cells and adipocytes supported the survival and proliferation of B-precursor ALL cell lines (Fig. 4). Importantly, we find that survival and proliferation of PDX ALL samples are differentially supported by stromal cells and adipocytes: stromal cells support the survival and proliferation of PDX cells significantly better than their adipocyte counterparts (Fig. 5). MS5 cells are long-known for their ability to support the growth of hemopoietic stem cells in vitro [17]. Since their initial development in 1989, they have become an important tool in the characterization of the BMM. For example, with the use of MS5 cells, stanniocalcin 1 was just recently identified as an important MSC-derived factor limiting the proliferation of hematopoietic stem cells in AML [29]. Although MS5 cells are of murine origin, they resemble primary human MSCs in terms of their ability to form spheroids, and they do so even with higher reliability than primary human MSCs [30]. Recently, a 3D model system of BMAd was established using primary murine and human MSCs [31]. Thus, our 2D MS5-derived co-culture system could even be developed further into a 3D spheroid model system. It is worth emphasizing that we observed differential survival and proliferation behavior of ALL cell lines and, in particular, of PDX cells on MS5-derived stromal cells and adipocytes. This suggests a large heterogeneity of leukemia samples with respect to their ability to adapt to different microenvironments, which could represent a key contributor to the risk of recurrence. Adipocytes can preserve leukemia cells from the cytotoxic effects of chemotherapy [5, 6, 9, 11–14]. Inhibition of proliferation in co-culture with adipocytes has been described to render ALL cells more resistant to cell cycle-dependent chemotherapeutic agents [6, 9]. Furthermore, several studies have shown that adipocytes can protect ALL cells from cytotoxicity in vitro in different ways, for example, by metabolizing anthracyclines into less active metabolites [13, 14]. We were therefore surprised to see that co-culture with adipocytes using our model system did not rescue ALL cell lines from daunorubicin-induced cell death, but on the contrary, even enhanced it (Fig. S8A). We were able to confirm this in experiments with PDX cells (Fig. 5D), a second anthracycline, doxorubicin (Fig. 5E), and dexamethasone (Fig. 5F). This data highlights once again the importance of well-controlled culture conditions—which we established in our work—for studying the role of adipocytes in ALL.

We propose that our model system could be applied to investigate interaction routes between leukemia cells and stromal cells or adipocytes by performing, e.g., bulk or single-cell RNA sequencing, proteomic analyses of co-culture supernatants, or CRISPR-Cas9-mediated knockout of candidate genes in stromal cells or adipocytes critical for leukemia cell survival. Furthermore, the model system could also be used as a drug screening platform to determine response to therapy in adipocyte-rich versus adipocyte-poor bone marrow microenvironments.

## Methods

### Antibodies

PPARγ (rabbit polyclonal; R&D Systems; 2443; 1:1000), Adiponectin (rabbit polyclonal; GeneTex; GTX112777; 1:1000), and ERK (rabbit; Cell Signaling; 9102; 1:2000).

### Cell lines

MS5 cells are available from DSMZ and cultured in alpha-MEM (Gibco, Thermo Fisher Scientific) supplemented with 10% FCS (Gibco, Thermo Fisher Scientific). 3T3-L1 cells are available from ATCC and cultured in DMEM (Gibco, Thermo Fisher Scientific) supplemented with 10% FCS and 2% L-glutamine (Gibco, Thermo Fisher Scientific). MS5 and 3T3-L1 cells were not authenticated.

B-precursor ALL cell lines Nalm6 (DSMZ no. ACC 128; Species: human), Reh (DSMZ no. ACC 22; Species: human), RS4;11 (DSMZ no. ACC 508; Species: human), RCH-ACV (DSMZ no. ACC 548; Species: human), EU3 (also known as cell line 697; DSMZ no. ACC 42; Species: human), and KOPN-8 (DSMZ no. ACC 552; Species: human) were maintained in RPMI (Gibco, Thermo Fisher Scientific) supplemented with 10–20% FCS and 2% L-glutamine. Cell lines were tested negative for Mycoplasma using the Mycoplasma detection kit MycoALERT (Lonza), and the identity of leukemia cell lines was confirmed by short tandem repeat (STR) profiling.

### NOD/SCID/huALL

Patient samples were obtained after informed consent in accordance with the institution’s Ethical Review Board (Ulm University); propagation of samples in mice was approved by and carried out in accordance with the appropriate authority (Regierungspraesidium Tuebingen). Patient-derived xenograft samples were established by transplantation of patient-derived ALL cells into 6-week-old female NOD/SCID mice (NOD.CB17-Prkdc^scid^/NCrCrl; strain code 394, Charles River) as previously described [32]. After the onset of leukemia, mice were sacrificed, and leukemia cells were isolated from the spleen and used for further experiments in this study. Inclusion/exclusion criteria, randomization, blinding or power analysis were not deemed relevant for this study.

### Adipogenic differentiation

*MS5 cells*: 3.5 × 10^3^ MS5 cells were seeded into 96-well plates or 3 × 10^4^ into 12-well plates on day −3 in their usual culture medium. On day 0, Adipogenic Medium I containing DMEM/F12 medium (Gibco, Thermo Fisher Scientific) supplemented with 10% FCS, 33 µM biotin (Sigma-Aldrich), 17 µM pantothenate (Sigma-Aldrich), 10 µg/ml transferrin, 20 nM insulin, 100 nM cortisol, 0.2 nM triiodothyronine (T3), 25 nM dexamethasone, 250 µM 3-isobutyl-1-methylxanthin (IBMX) and 2 µM rosiglitazone were added to the cells. On day 4 of adipogenic differentiation, Adipogenic Medium I was replaced by Adipogenic Medium II, consisting of DMEM/F12 medium supplemented with 10% FCS, 33 µM biotin, 17 µM pantothenate, 10 µg/ml transferrin, 20 nM insulin, 100 nM cortisol, and 0.2 nM T3. MS5 cells are considered to be fully differentiated adipocytes between days 10 and 14 of adipogenic differentiation.

*3T3-L1 cells*: See Supplementary Information.

### Serum-free and chemically defined leukemia cell-adipocyte co-culture system

MS5 cells were differentiated into adipocytes according to the differentiation protocol as outlined above. To assess the effects of their stromal counterparts, MS5 cells were seeded 3 days prior to the start of co-culture (usually on day 11 of adipogenic differentiation). On day 14 of adipogenic differentiation, medium was removed from both adipocytes and stromal cells, and cells were carefully washed twice with PBS prior to the addition of plain DMEM/F12 medium.

For co-culture experiments, B-precursor ALL cells were counted and stained with CellTrace Violet (CT) at a concentration of 1 µM according to the manufacturer’s instructions. CT positivity of PDX cells was confirmed by FACS directly after the staining procedure, and propidium iodide (PI) positivity was determined in parallel (day 0). Next, 2 × 10^4^ (for B-precursor ALL cell lines; ratio of leukemia cells to stromal cells/adipocytes = 2 to 1) or 1 × 10^5^ (for PDX samples; ratio of leukemia cells to stromal cells/adipocytes = 10 to 1) CT-positive leukemia cells were seeded either in plain DMEM/F12 medium or together with adipocytes or stromal cells.

At the time point of analysis, the medium was harvested and transferred to a deep well plate. Prior to adding trypsin to the wells for 5 min to detach cells, wells were rinsed once with PBS. FCS was subsequently added to inactivate trypsin, and cells were collected in a deep well plate. Cells were stained with PI at a final concentration of 1 µg/ml, and samples were measured by automated sampling at the Attune NxT cytometer (Thermo Fisher Scientific).

### Western blot

Cells were washed once with PBS prior to lysis in lysis buffer containing 10 mM Tris–HCl (pH 7.5), 150 mM NaCl, 2 mM EDTA, 1% Triton X-100, and 10% Glycerol supplemented with protease inhibitor (Roche). Cells were harvested using a cell scraper, and lysis was continued on ice for 20 min. Lysates were then centrifuged for 30 min at 14,000 rpm at 4 °C. Cleared lysates were stored at −20 °C until further analysis. Protein concentration was determined using the BCA assay (Thermo Fisher Scientific). Bolt LDS sample buffer (Invitrogen) and reducing agent (Invitrogen) were added to the adjusted lysates. Lysates were denatured at 90 °C for 10 min before separation by SDS–PAGE (NuPAGE). Membranes were incubated with primary antibodies at 4 °C overnight or for 1 h at room temperature. Washing of membranes was performed in 1 x TBS containing 0.05% Tween-20 (Sigma-Aldrich) for 3 × 10 min before incubation with the secondary antibody for 1 h at room temperature.

Uncropped Western blots can be found in the Supplemental Material.

### Oil Red O staining

Cells were washed once with PBS prior to incubation with 4% paraformaldehyde (PFA) at room temperature for 10 min. Samples were then stored at 4 °C until further analysis. For staining with Oil Red O, cells were washed with 60% isopropanol prior to the addition of Oil Red O staining solution (6 parts of Oil Red O stock solution (3.5 g Oil Red O in 1 l isopropanol) were mixed with 4 parts of distilled water). After incubation at room temperature for 10 min, the staining solution was removed, and cells were washed at least 3 times with H_2_O. Pictures were taken with the Keyence BZ-9000 microscope or the Incucyte S3.

### RNA isolation and qRT-PCR

RNA was isolated with the RNeasy Mini Kit (Qiagen) or the Quick-RNA Miniprep Kit (Zymo Research) according to the manufacturer’s instructions. cDNA was synthesized using SuperScript II Reverse Transcriptase (Thermo Fisher Scientific) with random primers (Thermo Fisher Scientific). qRT-PCR was performed using iTaq Universal SYBR Green Supermix (Bio-Rad) on a Bio-Rad CFX Connect Real-Time PCR Detection System using the following protocol: 95 °C for 30 s, then 40 cycles of 95 °C for 5 s followed by 60 °C for 30 s.

The primer sequences used can be found in Supplementary Table 1.

### Cell viability assay

Cell viability was determined using CellTiter-Glo® assay (G7572, Promega) according to the manufacturer’s instructions.

### Transwell assay

MS5-derived stromal cells or adipocytes were prepared as outlined under “Serum-free and chemically defined leukemia cell-adipocyte co-culture system”. 2 × 10^5^ B-precursor ALL cells labeled with 1 µM CT Violet were seeded in DMEM/F12 medium into transwell inserts (Falcon, pore size 0.4 µm) over medium, MS5-derived stromal cells, or adipocytes. After 7 days, cells were harvested, stained with PI at a final concentration of 1 µg/ml, and measured at the Attune NxT cytometer.

### Seahorse extracellular flux analysis

*Leukemia cells*: 5 × 10^5^ leukemia cells were seeded into transwell inserts (Falcon, pore size 0.4 µm) over medium, MS5-derived stromal cells, or adipocytes in DMEM/F12 medium. After 96 h, cells were harvested, counted, and seeded at 1 × 10^5^ cells/well into Seahorse cell culture plates, which had been precoated with Cell-Tak (Corning, 22.4 µg/ml). For attachment of leukemia cells, Seahorse cell culture plates were centrifuged (200×*g*, 1 min, deceleration 0) prior to measurement at the XFe96 Extracellular Flux Analyzer (Agilent). Uncoupled (proton leak) respiration was profiled by injecting 1.5 µM oligomycin (an ATP synthase inhibitor), and full substrate oxidation capacity was determined by injecting 0.75 µM carbonylcyanide-*p*-trifluoromethoxyphenylhydrazone (FCCP, a chemical uncoupler). Non-mitochondrial respiration was determined by injecting 0.5 µM antimycin A and 0.5 µM rotenone (ETC inhibitors). Data were normalized by staining cells with Janus Green dye prior to analysis on a Tecan multimode reader (Tecan).

*MS5-derived stromal cells and adipocytes*: 2.5 × 10^3^ MS5-derived stromal cells were seeded into Seahorse cell culture plates and further differentiated or not into adipocytes. Once fully differentiated, cells were incubated with conditioned medium of B-precursor ALL cell lines. After 24 h, cells were analyzed at the XFe96 Extracellular Flux Analyzer. To interrogate mitochondrial parameters as outlined above, the following injector concentrations were used: oligomycin 1 µM, FCCP 0.5 µM (adipocytes) or 2 µM (stromal cells), rotenone 0.5 µM, antimycin A 0.5 µM. Data were normalized by staining cells with Janus Green dye prior to analysis on a Tecan multimode reader (Tecan).

ATP production rates were calculated from oxygen consumption rate (OCR) and extracellular acidification rate (ECAR) data, assuming a P/O ratio of 2.75.

### Generation of leukemia cell-conditioned medium

For the generation of conditioned medium, B-precursor ALL cell lines were washed twice with PBS, counted, and seeded at 1 × 10^6^ cells/ml in plain DMEM/F12 medium. After 72 h, cells were harvested, centrifuged at 2500 rpm for 10 min, and stored at −20 °C until further use.

### Statistical analysis

No statistical methods were used to pre-determine sample size for in-vitro experiments. In-vitro experiments were performed at least three times. Data were analyzed using GraphPad Prism software (version 10.4.1, San Diego, CA). The statistical tests applied are provided for each figure in the respective figure legend.

## Supplementary information

uncropped Western blots

Supplementary Information

## Data Availability

The data generated in this study are available upon request from the corresponding author.
