# Supplementary material for: Bridging the marrow: a co-culture-platform of leukemia cells and MS5-derived stromal cells or adipocytes
Source: Cell Death Discov. 2025 Aug 5;11:366. doi: 10.1038/s41420-025-02631-5 (PMC12325993; doi:10.1038/s41420-025-02631-5)

Uncropped Western Blots

Bridging the Marrow: A Co-Culture-Platform of Leukemia Cells and MS5-derived Stromal Cells or Adipocytes

Julia Zinngrebe, Elena Dorothea Brenner, Ferdinand Schlichtig, Ulrich Stifel, Daniel Tews, Jana Falk, Dominik Schlotter, Rahel Fitzel, Lüder-Hinrich Meyer, Klaus-Michael Debatin, Pamela Fischer-Posovszky

Figure 1D

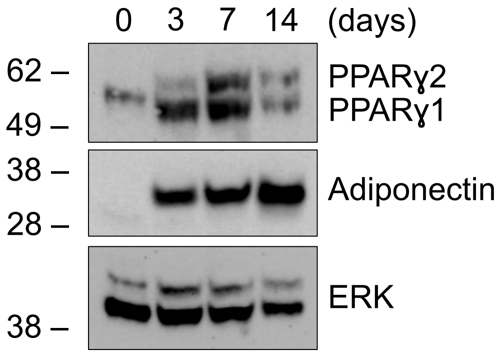

PPARɣ1/2

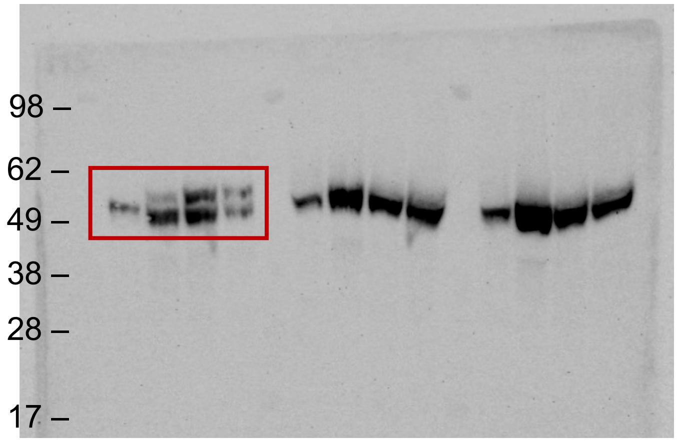

Adiponectin

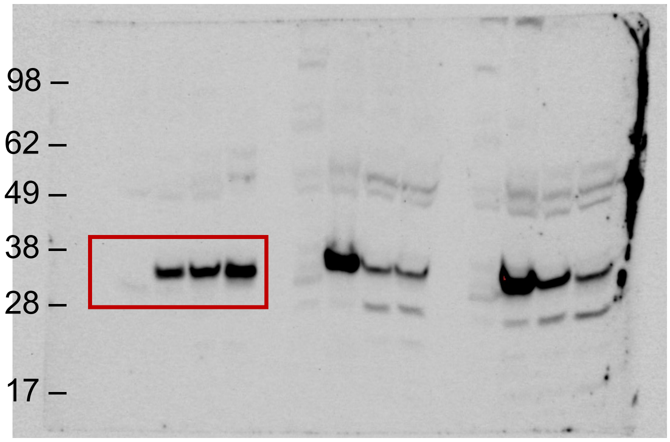

ERK

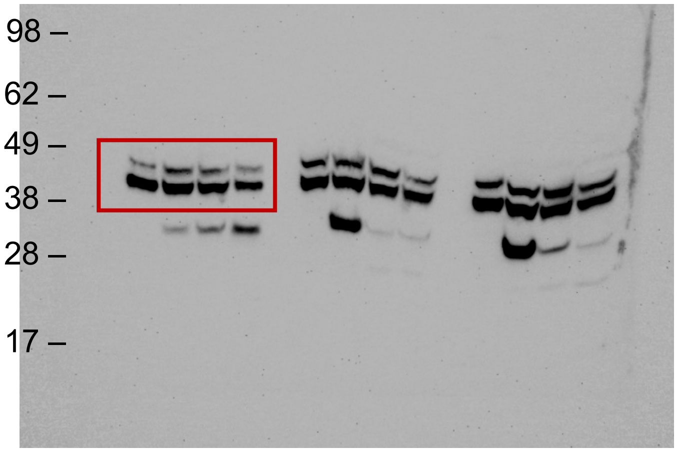

Figure 3C

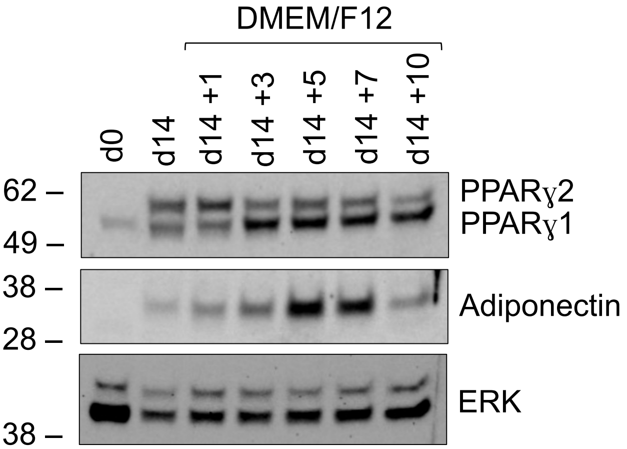

PPARγ1/2

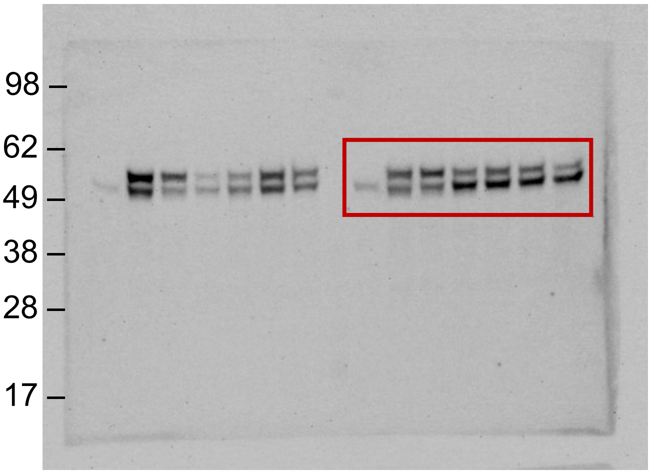

Adiponectin

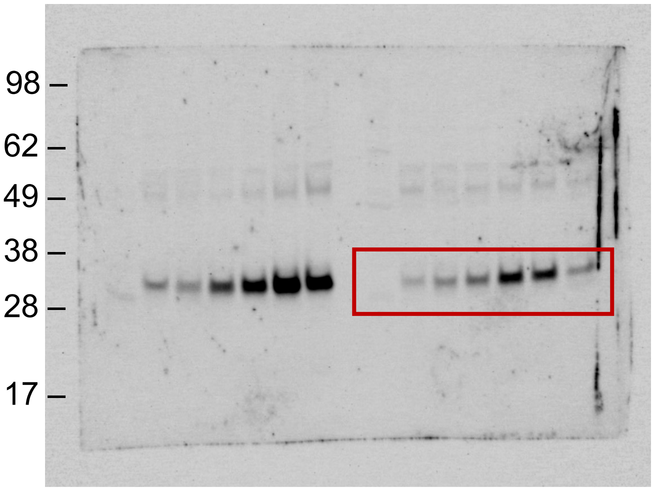

ERK

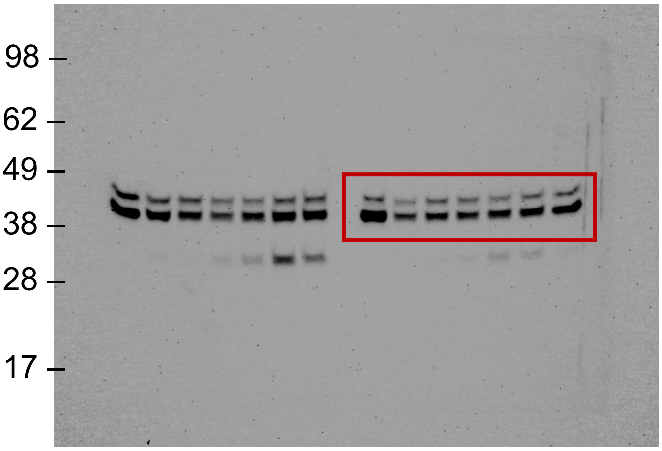

Figure S1G

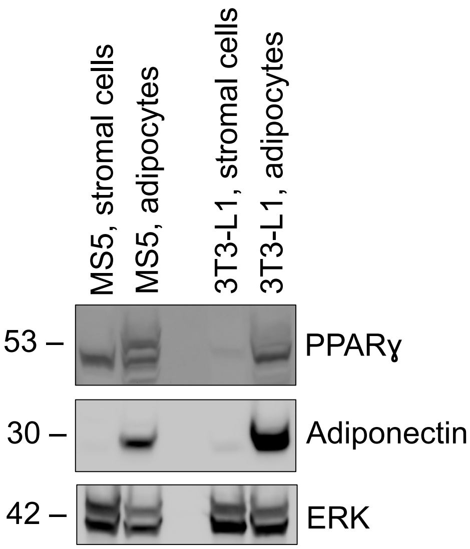

PPARɣ1/2

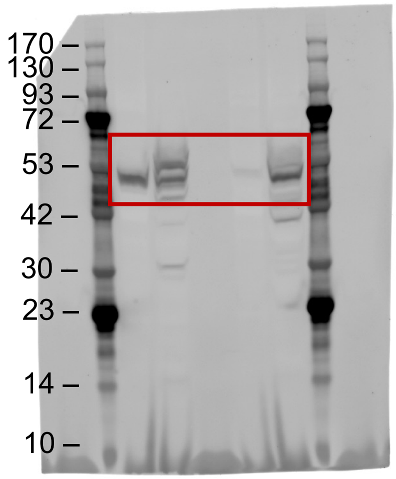

Adiponectin

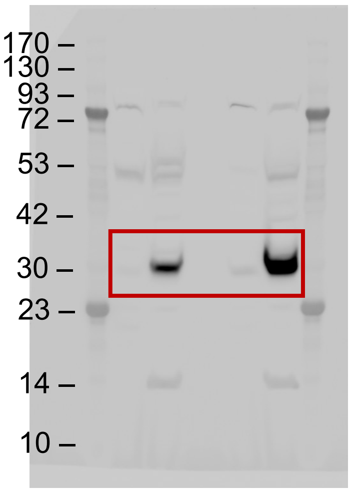

ERK

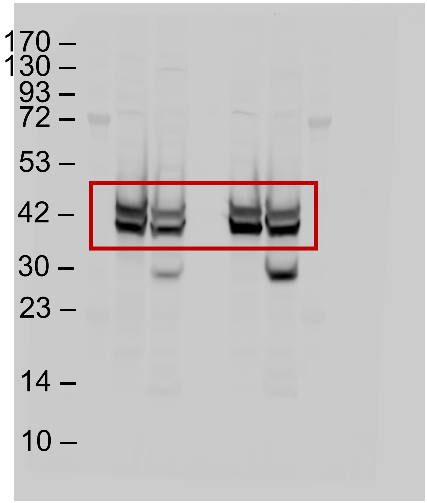

Supplement: Supplementary file 1 — uncropped Western blots [file 41420_2025_2631_MOESM1_ESM.pdf]
