# Supplementary material for: Bridging the marrow: a co-culture-platform of leukemia cells and MS5-derived stromal cells or adipocytes
Source: Cell Death Discov. 2025 Aug 5;11:366. doi: 10.1038/s41420-025-02631-5 (PMC12325993; doi:10.1038/s41420-025-02631-5)
Supplement: Supplementary file 2 — Supplementary Information [file 41420_2025_2631_MOESM2_ESM.pdf]

Supplementary Information

Bridging the Marrow: A Co-Culture-Platform of Leukemia Cells and MS5-derived Stromal Cells or Adipocytes

Julia Zinngrebe [1], Elena Dorothea Brenner [1], Ferdinand Schlichtig [1], Ulrich Stifel [1], Daniel Tews [1,2], Jana Falk [1], Dominik Schlotter [1], Rahel Fitzel [1], Lüder-Hinrich Meyer [1], Klaus-Michael Debatin [1, 3], Pamela Fischer-Posovszky [1, 3, #]

[1]Department of Pediatrics and Adolescent Medicine, Ulm University Medical Center, 89075 Ulm, Germany

[2]Division of Pediatric Endocrinology and Diabetes, Department of Pediatrics and Adolescent Medicine, Ulm University Medical Center, 89075 Ulm, Germany

[3]German Center for Child and Adolescent Health (DZKJ), partner site Ulm, Ulm, Germany

[#]Corresponding author: Professor Pamela Fischer-Posovszky, PhD

pamela.fischer@uniklinik-ulm.de

Supplementary Figures

Figure S1

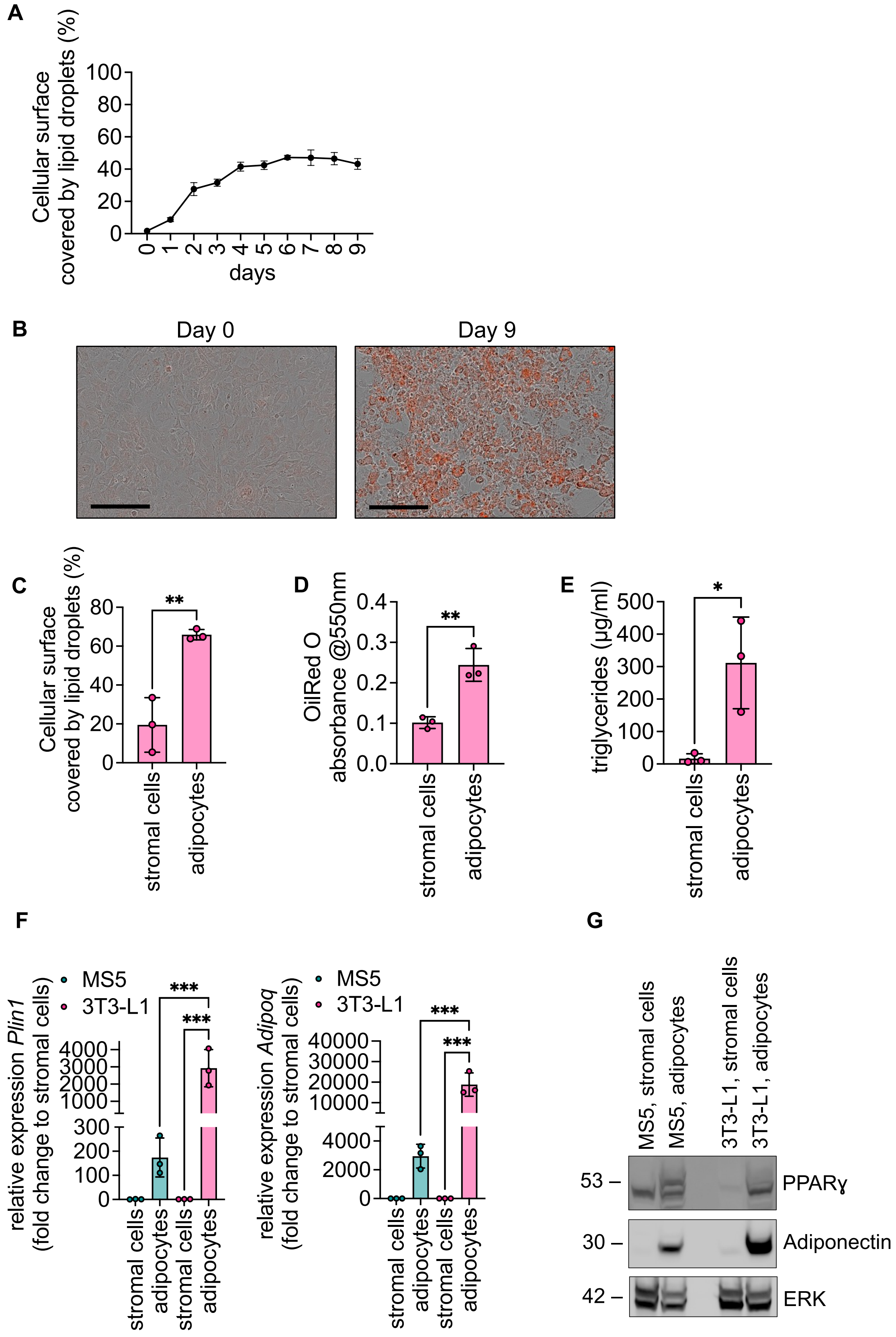

Fig S1. Adipogenic differentiation of MS5 and 3T3-L1 cells.

(A) Cellular surface covered by lipid droplets during the course of adipogenic differentiation of MS5 cells is shown. (B – D) 3T3-L1 stromal cells (day 0 of adipogenic differentiation) and adipocytes (day 9 of adipogenic differentiation) were stained with OilRed O and imaged in the Incucyte S3 (B). Cellular surface covered by lipid droplets (C) and quantification of OilRed O staining are shown (D). (E) Triglycerides were determined in stromal cells (day 0) and adipocytes (day 9). (F – G) mRNA (F) and protein (G) expression of MS5 and 3T3-L1 stromal cells (day 0) and adipocytes (day 9) is depicted. Data are presented as mean ± SD from three independent experiments performed in at least triplicates. Unpaired t-test (C, D, E) or ordinary one-way ANOVA with Bonferroni's multiple comparisons test (F), *, $P < 0.05$; **, $P < 0.01$; ***, $P < 0.001$. Images (B, G) are representative of three independent experiments. Scale bar equates to 200 μm.

Figure S2

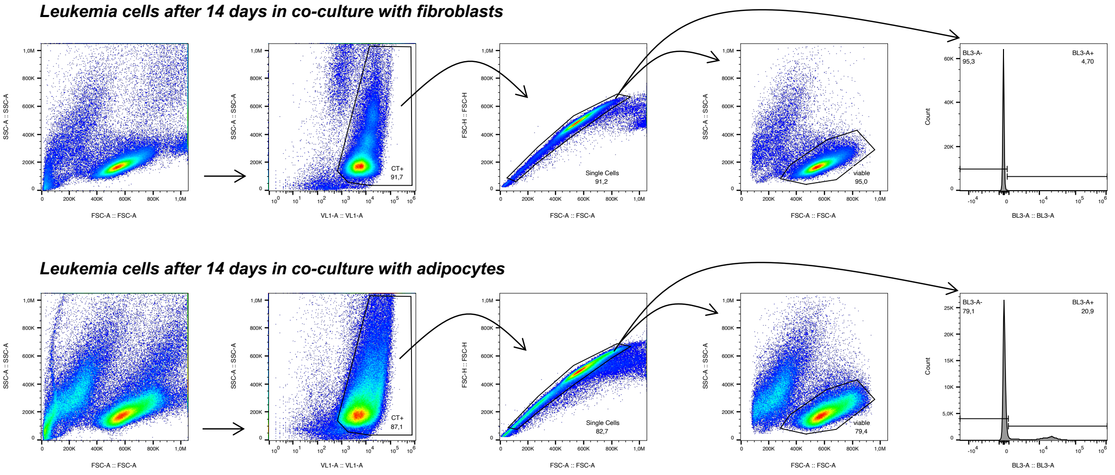

VL1: CellTrace Violet (CT)
BL3: Propidium Iodide (PI)

Fig S2. Staining with CellTrace Violet allows robust discrimination of leukemia cells from both, stromal cells or adipocytes.

Gating strategy of leukemia cells in co-culture with fibroblasts (upper panel) or adipocytes (lower panel) is depicted.

Figure S3

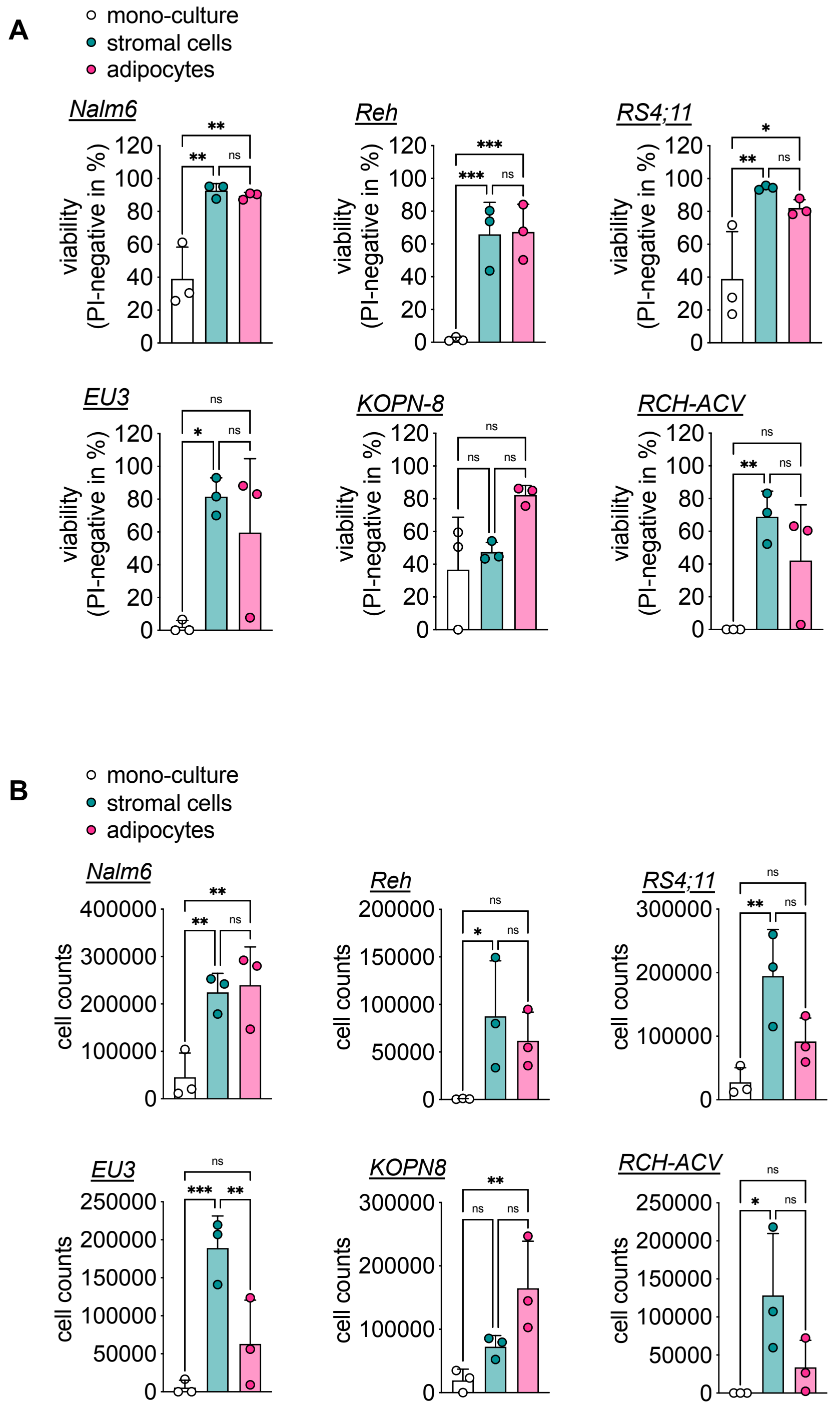

Fig S3. MS5-derived stromal cells and adipocytes can maintain survival and proliferation of B-precursor ALL cell lines.

(A – B) B-precusor ALL cell lines Nalm6, Reh, RS4;11, EU3, KOPN-8, and RCH-ACV were cultured alone or together with stromal cells or adipocytes derived from MS5 cells. Cell

viability (A) or cell counts of the number of viable cells per well (B) were determined after 7 days. Data are presented as mean + SD from three independent experiments performed in triplicates; dots represent individual experiments. *, $P < 0.05$; **, $P < 0.01$; ***, $P < 0.001$; ****, $P < 0.0001$; one-way ANOVA with Tukey’s multiple comparisons test.

Figure S4

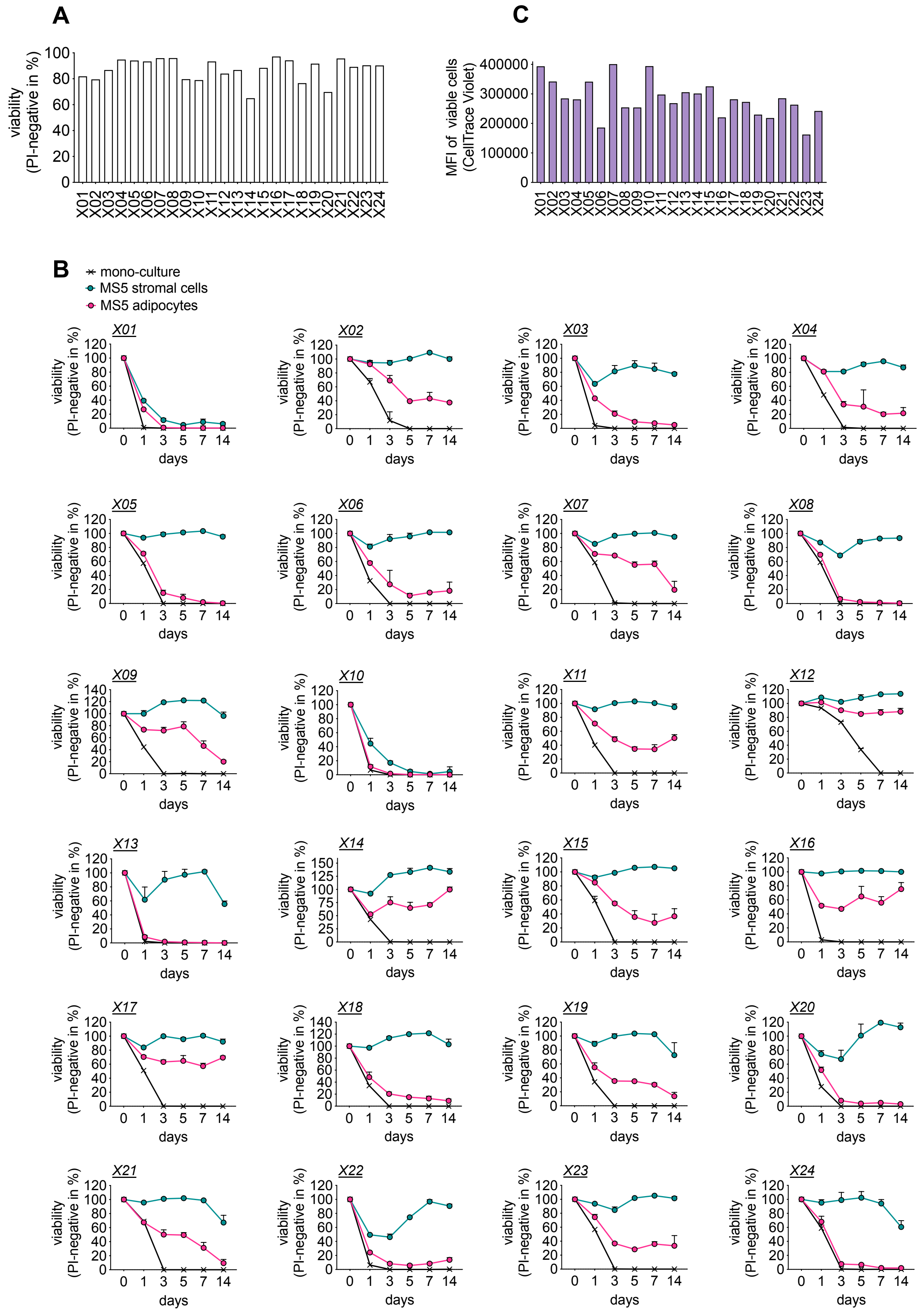
A
viability
(PI-negative in %)
X01 X02 X03 X04 X05 X06 X07 X08 X09 X10 X11 X12 X13 X14 X15 X16 X17 X18 X19 X20 X21 X22 X23 X24
C
MFI of viable cells
(CellTrace Violet)
X01 X02 X03 X04 X05 X06 X07 X08 X09 X10 X11 X12 X13 X14 X15 X16 X17 X18 X19 X20 X21 X22 X23 X24
B
mono-culture
MS5 stromal cells
MS5 adipocytes
X01
X02
X03
X04
X05
X06
X07
X08
X09
X10
X11
X12
X13
X14
X15
X16
X17
X18
X19
X20
X21
X22
X23
X24
viability
(PI-negative in %)
days

Fig S4. Co-culture of MS5-derived stromal cells or adipocytes with PDX B-precusor ALL cells.

(A) Viability of PDX B-precursor ALL samples (n=24) on day 0. (B) Individual PDX B-precursor ALL samples (n=24) were cultured alone or together with stromal cells or adipocytes derived from MS5 cells. Viability as determined by propidium-iodide (PI)-negative cells on different days after seeding is shown. Data are presented as mean + SD of samples measured in triplicates. (C) Mean fluorescence intenstity (MFI) of CellTrace-labelled PDX samples on day 0 is depicted.

Figure S5

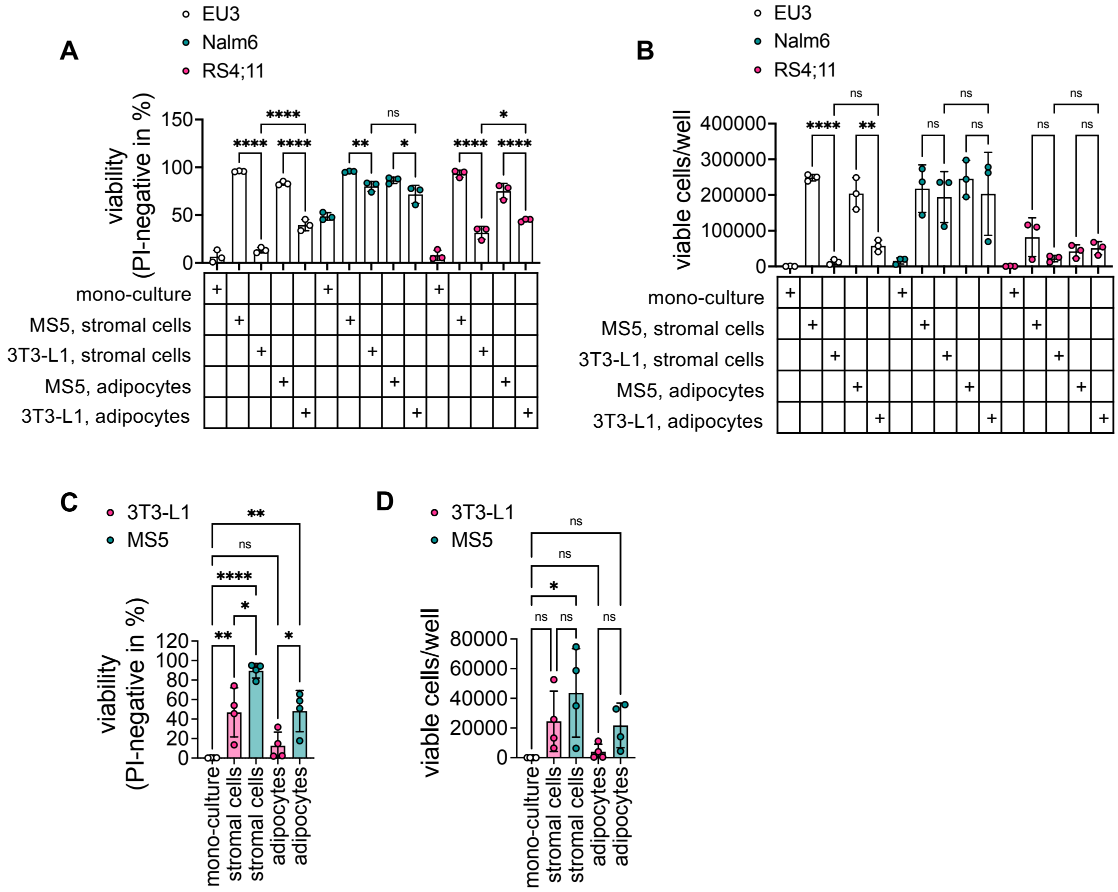

Fig S5. Co-culture of leukemia cells with MS5-derived or 3T3-L1-derived stromal cells or adipocytes. (A – B) B-precusor ALL cell lines EU3, Nalm6 and RS4;11 were cultured alone or together with MS5-derived or 3T3-L1-derived stromal cells or adipocytes. Cell viability (A) or viable cells/well (B) were determined after 7 days. Data are presented as mean ± SD from three independent experiments performed in five technical replicates; dots represent individual experiments. (C – D) PDX B-precursor ALL samples X07, X12, X14, and X17 were cultured alone or together with 3T3-L1- or MS5-derived stromal cells or adipocytes. Viability (C) and the number of viable cells/well (D) were determined after 7 days in culture. Data are presented as mean ± SD from four individual PDX samples performed in three technical replicates; dots represent individual PDX samples. Ordinary one-way ANOVA with Šidák's multiple comparisons test; *, $P < 0.05$; **, $P < 0.01$; ****, $P < 0.0001$.

Figure S6

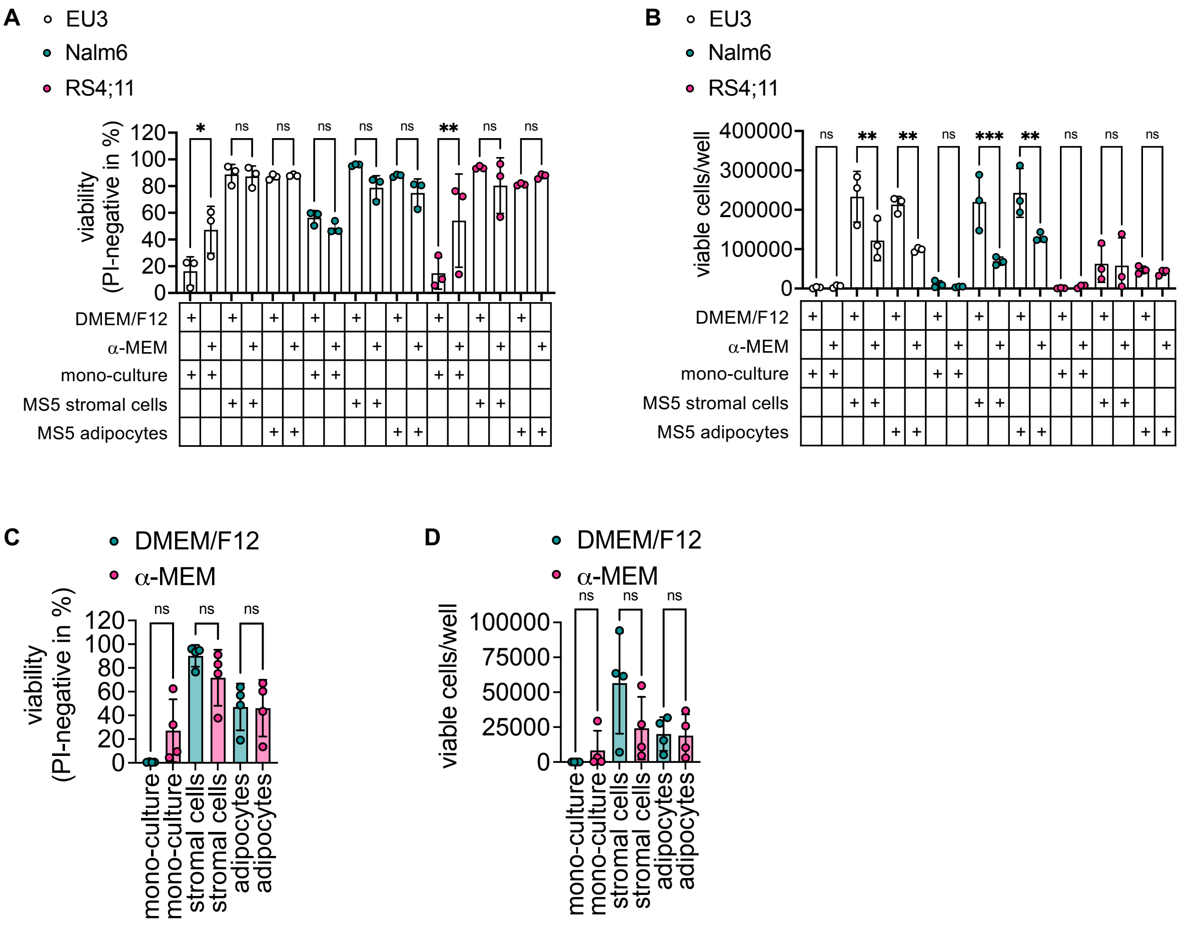

Fig S6. Co-culture of leukemia cells with MS5-derived stromal cells or adipocytes cultured in DMEM/F12 or alpha-MEM. (A – B) B-precusor ALL cell lines EU3, Nalm6 and RS4;11 were cultured alone or together with MS5-derived stromal cells or adipocytes in either DMEM/F12 or alpha-MEM. Cell viability (A) or viable cells/well (B) were determined after 7 days. Data are presented as mean ± SD from three independent experiments performed in five technical replicates; dots represent individual experiments. (C – D) PDX B-precursor ALL samples X07, X12, X14, and X17 were cultured alone or together with MS5-derived stromal cells or adipocytes in either DMEM/F12 or alpha-MEM. Viability (C) and the number of viable cells/well (D) were determined after 7 days in culture. Data are presented as mean ± SD from four individual PDX samples performed in three technical replicates; dots represent individual

PDX samples. Ordinary one-way ANOVA with Šidák's multiple comparisons test; *, $P < 0.05$; **, $P < 0.01$; ***, $P < 0.001$.

Figure S7

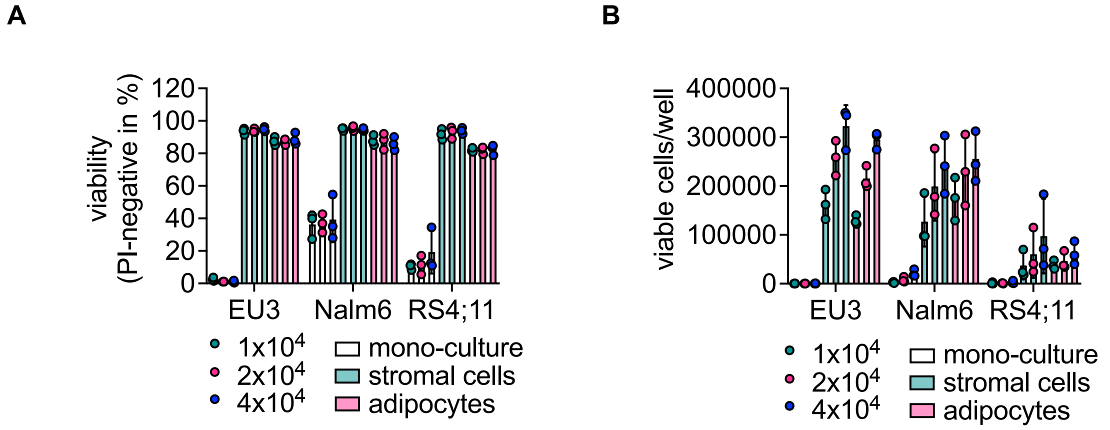

Absolute number of leukemia cells	Absolute number of stromal cells/adipocytes	Ratio of leukemia cells : stromal cells/adipocytes
1x10^{4}	1x10^{4}	1:1
2x10^{4}	1x10^{4}	2:1
4x10^{4}	1x10^{4}	4:1

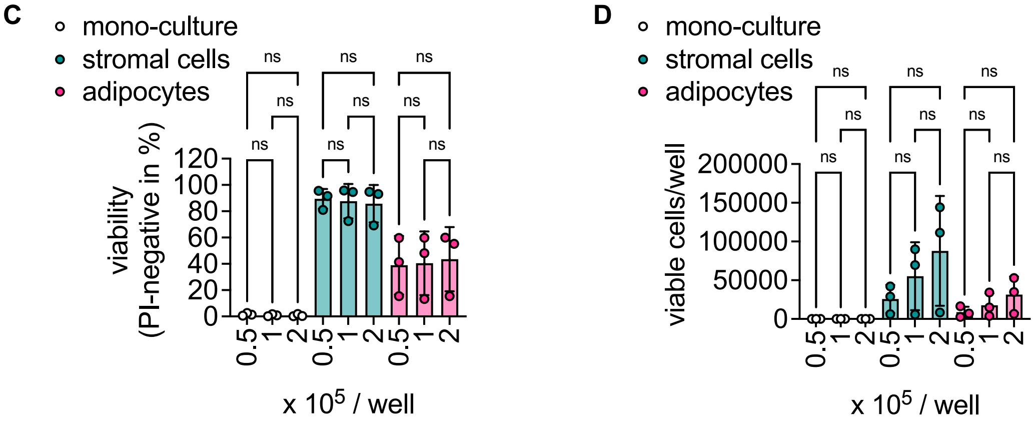

Absolute number of leukemia cells	Absolute number of stromal cells/adipocytes	Ratio of leukemia cells : stromal cells/adipocytes
0.5x10^{5}	1x10^{4}	5:1
1x10^{5}	1x10^{4}	10:1
2x10^{5}	1x10^{4}	20:1

Fig S7. Co-culture of MS5-derived stromal cells or adipocytes and different densities of leukemia cells. (A – B) 1x10^{4}, 2x10^{4} or 4x10^{4} EU3, Nalm6 or RS4;11 cells were cultured alone or together with MS5-derived stromal cells or adipocytes. Cell viability (A) or viable cells/well (B) were determined after 7 days. Data are presented as mean ± SD from three

independent experiments performed in three technical replicates; dots represent individual experiments. (C – D) 0.5x10^5, 1x10^5 or 2x105 PDX B-precursor ALL cells (*n*=3; X12, X14, and X17) were cultured alone or together with MS5-derived stromal cells or adipocytes. Viability (C) and the number of viable cells/well (D) were determined after 7 days in culture. Data are presented as mean ± SD from three individual PDX samples performed in three technical replicates; dots represent individual PDX samples. Ordinary one-way ANOVA with Šidák's multiple comparisons test.

Figure S8

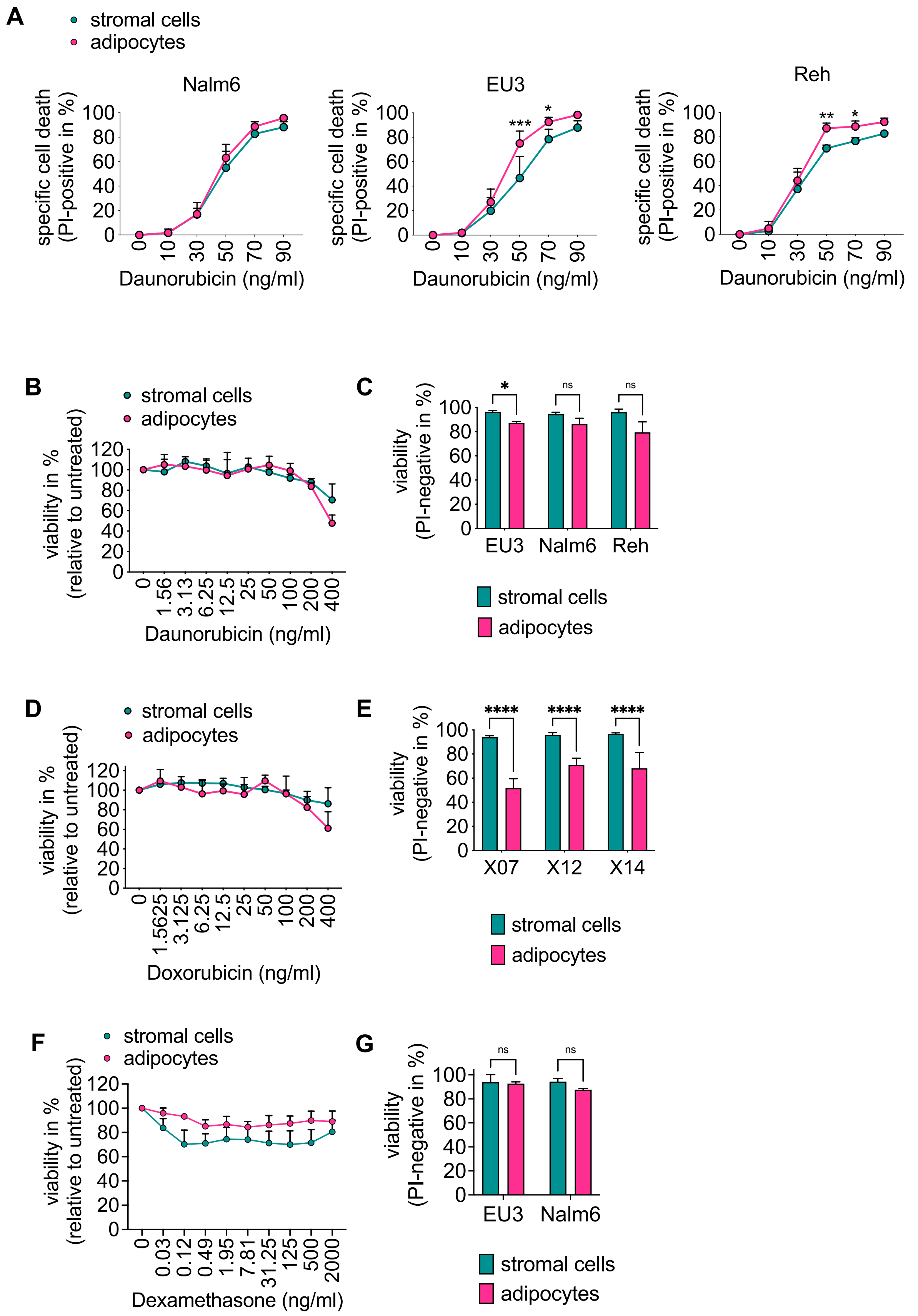
A
stromal cells
adipocytes
Nalm6
EU3
Reh
specific cell death (PI-positive in %)
***
*
**
*
Daunorubicin (ng/ml)
B
stromal cells
adipocytes
viability in % (relative to untreated)
Daunorubicin (ng/ml)
C
viability (PI-negative in %)
*
ns
ns
EU3
Nalm6
Reh
stromal cells
adipocytes
D
stromal cells
adipocytes
viability in % (relative to untreated)
Doxorubicin (ng/ml)
E
viability (PI-negative in %)
****
****
****
X07
X12
X14
stromal cells
adipocytes
F
stromal cells
adipocytes
viability in % (relative to untreated)
Dexamethasone (ng/ml)
G
viability (PI-negative in %)
ns
ns
EU3
Nalm6
stromal cells
adipocytes

Fig S8. MS5-derived fibroblasts and adipocytes are resistant to anthracycline- or dexamethasone-induced cell death.

(A) Leukemia cell lines Nalm6, EU3 and Reh were stimulated with increasing concentrations of daunorubicin as indicated in co-culture with either MS5-derived stromal cells or adipocytes. Cell death was assessed by PI positivity at the FACS after 96 hours. (B, D, F) MS5-derived stromal cells or adipocytes were treated with increasing concentrations of daunorubicin (B), doxorubicin (D) or dexamethasone (F). Viability was determined after seven days using CellTiter-Glo®. (C, E, G) Corresponding viability of untreated leukemia cells from Fig S8A (C), Fig 5D and E (E) or Fig 5F (G). Data are presented as mean + SEM (A) or + SD (B – G) from at least three independent experiments performed in triplicates (A – D, F and G) or three technical replicates of the individual PDX B-precursor ALL samples (E). *, $P < 0.05$; **, $P < 0.01$; ***, $P < 0.001$; ****, $P < 0.0001$; two-way ANOVA with Šídák's multiple comparisons test (A, C, E, G).

Supplementary Methods

Determination of cellular surface covered by lipid droplets

The cellular surface covered by lipid droplets was determined in an automated manner by using the live-cell analysis system Incucyte S3 (Sartorius) followed by analysis with the Incucyte 2019B Rev2 software and the basic analyzer software module. Analysis was performed on at least 3 different wells of a 12-well plate with MS5 or 3T3-L1 cells undergoing adipogenic differentiation. At least 4 different images were taken per well with a 10x or 20x magnification. Analysis was performed on phase contrast images to determine (i) the area of the well covered by lipid droplets, and (ii) the area of the well covered by cells. From this the percentage of the cellular surface covered by lipid droplets was calculated ((i) divided by (ii) multiplied by 100).

Adipogenic differentiation of 3T3-L1 cells

3T3-L1 cells were differentiated based on a previoulsy published differentiation protocol (1). In brief, 3.5 x 10^3 3T3-L1 cells were seeded into each well of a 96-well plate or 4 x 10^4 into each well of a 12-well plate on day minus 3 of adipogenic differentiation in their usual culture medium, i.e. DMEM (Gibco) supplemented with 10% FCS and 2% L-Glutamine. On day 0 of adipogenic differentiation, the culture medium was changed to differentation medium (DMEM plus 10% FCS and 2% L-Glutamine supplemented with 500 µM IBMX, 5 µg/ml insulin, 5 µM dexamethasone, 125 µM indomethacin, 1 nM T3 and 1 µM rosiglitazone). On day 4 of adipogenic differentiation, the differentiation medium was exchanged by maturation medium (DMEM plus 10% FCS and 2% L-Glutamine supplemented with 5 µg/ml insulin and 1 µM rosiglitazone). The maturation medium was replaced every three to four days by fresh maturation medium. 3T3-L1 cells are considered to be mature and fully differentiated adipocytes after 8 - 10 days of adipogenic differentiation (2, 3).

Supplementary Table 1. Primer sequences

Adipoq	gttcctcttaatcctgcccagtcatgcc (f), ggaccaagaagacctgcatctcctttctc (r)
Fabp4	cgatgattacatgaaagaagtgggagtggg (f), tatgatgctcttcaccttcctgtcgtctg (r)
Glut4	tgagctgaaggatgagaaacggaagttgga (f), ctaagagcaccgagaccaacgtgaagacc (r)
Hprt	gctggtgaaaaggacctc (f), cacaggactagaacacct (r)
Il6	gatggatgctaccaaactgga (f), tctgaaggactctggctttg (r)
Mcp1	aggtccctgtcatgcttctg (f), ggatcatcttgctggtgaa (r)
Plin	caatgcctatgagaagggtgtacagggtg (f), caactcattggcagctgtgaactgggtg (r)
Pparg	gaccactcgcattcctttgacatcaagcc (f), tgatcgcactttggtattcttggagcttcag (r)

References

1. Caratti G, Stifel U, Caratti B, Jamil AJM, Chung KJ, Kiehntopf M, et al. Glucocorticoid activation of anti-inflammatory macrophages protects against insulin resistance. Nat Commun. 2023;14(1):2271.
2. Kaczmarek I, Suchy T, Strnadova M, Thor D. Qualitative and quantitative analysis of lipid droplets in mature 3T3-L1 adipocytes using oil red O. STAR Protoc. 2024;5(2):102977.
3. Sun W, Yu Z, Yang S, Jiang C, Kou Y, Xiao L, et al. A Transcriptomic Analysis Reveals Novel Patterns of Gene Expression During 3T3-L1 Adipocyte Differentiation. Front Mol Biosci. 2020;7:564339.
